# Cellulose as a photocatalyst support material: extraction, structural features, and environmental applications

**DOI:** 10.3762/bjnano.17.44

**Published:** 2026-05-12

**Authors:** Yee Teng Lim, Nur Farhana Jaafar, Azizul Hakim Lahuri, Endang Tri Wahyuni

**Affiliations:** 1 School of Chemical Sciences, Universiti Sains Malaysia, 11800 USM Penang, Malaysia; 2 Department of Science and Technology, Universiti Putra Malaysia Bintulu Campus, Bintulu, Malaysia; 3 Chemistry Department, Faculty of Mathematic and Natural Sciences, Gadjah Mada University, Yogyakarta, 55281, Indonesia

**Keywords:** biomass waste, cellulose, green technology, photocatalysis, sustainable materials

## Abstract

Cellulose is the most abundant and renewable biopolymer found in nature, primarily derived from plant biomass. Its wide availability, low cost, biodegradability, and non-toxic nature make it a promising candidate for sustainable material development across various industries. This review focuses on the extraction and isolation of cellulose from different types of biomass waste, including agricultural residues, forestry byproducts, and industrial waste. Several conventional and advanced methods for cellulose extraction were also explored such as acid hydrolysis, enzymatic hydrolysis, oxidation, and mechanical or solvent-based techniques. Besides that, this review highlights the role of cellulose in photocatalytic applications, where its high surface area, structural porosity, and abundance of functional groups enable it to act as an effective support matrix for photocatalysts such as ZnO, TiO_2_, Cu_2_O, and various hybrid nanocomposites. Through stabilizing nanoparticles, preventing their aggregation and contributing to pollutant adsorption, cellulose enhances the overall efficiency of photocatalytic systems, improving its performance under a wide range of light sources. Moreover, cellulose-supported systems often show enhanced recyclability and mechanical durability, making them suitable for repeated use in wastewater treatment and environmental remediation. The relationship between cellulose’s structural properties and photocatalytic functionality offers a green and efficient solution to pressing environmental challenges. Overall, this review underscores the importance of cellulose not only as a raw material but also as an active component in next-generation, eco-friendly photocatalytic technologies.

## Introduction

The utilization of biomass has gained a lot of interest among researchers; biomass materials for extraction of biopolymers originate from plants, wood, and waste such as fruit peels and used paper products. Polysaccharides serve as the primary source of biopolymers, forming the main structural components of both woody and nonwoody materials, and they are composed of long carbohydrate chains that consist primarily of monosaccharide units. Unlike fossil-based alternatives, these natural polymers provide distinct advantages such as renewability, biodegradability, and biocompatibility, which make them particularly valuable for biomedical applications. Among polysaccharides, cellulose is the most abundant biodegradable polymer [1]. Many researchers have reported on the extraction of cellulose fiber from various sources such as walnut [2], date seeds [3], and corn husk [1].

**Figure 1:**
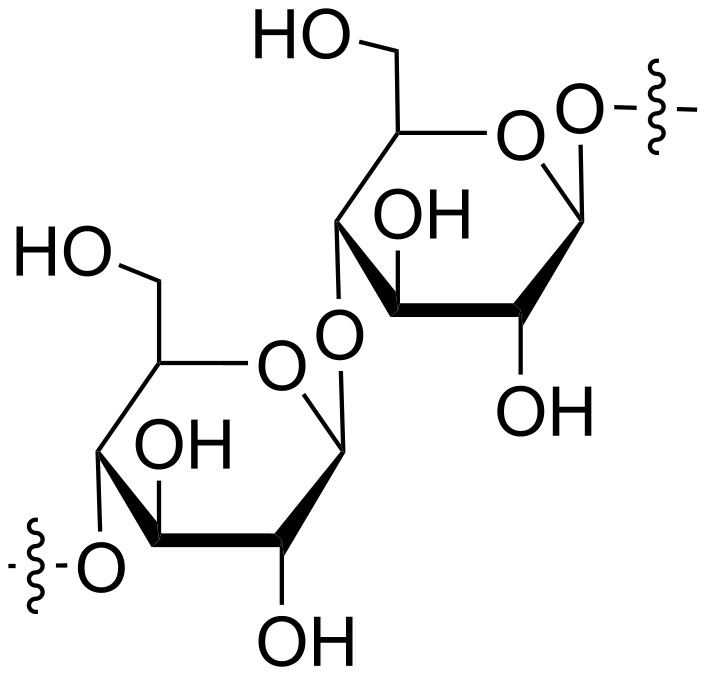
Structure of cellulose.

Cellulose (Figure 1) is a linear homopolysaccharide composed of repeating β-ᴅ-glucopyranose units that are linked together through β-1,4-glycosidic bonds, with each glucopyranose group carrying three free hydroxy groups that contribute to the unique reactivity and structural properties of the polymer. These hydroxy groups enable extensive hydrogen bonding between polymer strands; together with van der Waals forces, they drive the cellulose molecules to align and stack in parallel, ultimately giving rise to the formation of nanofibers. These nanofibers further assemble into larger cellulose microfibrils [4]. With the global cellulose production now exceeding hundreds of billions of tons annually and continuing to grow steadily, this abundant biopolymer stands out for its exceptional mechanical, physical, and chemical properties [5]. Cellulose occurs naturally in cotton and wood, where it has long provided essential resources for clothing and shelter. Also, through chemical and mechanical processing, pulp fibers can be extracted from these sources to serve as the primary raw material for paper production. Pulp fibers can undergo further mechanical and chemical treatments that transform them into nanocelluloses, an advanced class of materials that offer enhanced functionality and demonstrate superior properties compared to their bulk counterparts [6].

Nanocellulose is generally classified into two primary types and has recently gained significant attention due to its unique chemical and physical properties. The first type comes from acid treatment, and this form is called cellulose nanocrystals (CNCs). The second type, which results primarily from mechanical disintegration, is known as cellulose nanofibrils (CNFs) [7]. CNCs and CNFs differ mainly in their size and crystallinity, as CNFs contain both amorphous and crystalline cellulose chains and can extend to several micrometers in length. In contrast, CNCs exhibit high crystallinity, with lengths typically measuring below 500 nm. Both materials offer exciting possibilities for diverse applications [6].

Extracting cellulose from waste is crucial for its effective use. Several methods exist such as acid hydrolysis [3], enzymatic hydrolysis [8], green solvent-based extraction [9], and ionic liquid treatment [10], where each method has specific advantages and disadvantages. Acid hydrolysis is a traditional approach that helps preserve cellulose’s crystalline structure; however, it relies on non-biodegradable chemicals and generates acidic wastewater, which raises environmental concerns and complicates wastewater management [8]. As a result, researchers are exploring more sustainable alternatives. Enzymatic hydrolysis is an efficient and eco-friendly way to produce cellulose nanoparticles. This method breaks down cellulose fibers with precision under controlled conditions while using very little water and producing non-hazardous waste as well as potentially recovering and reusing the dissolved sugars [8]. Ionic liquids treatment serves as promising green alternatives to harmful organic solvents and their negligible vapor pressure makes them particularly attractive. Unlike traditional solvents, ionic liquids are non-flammable and possess unique properties that allow their effective use in multiple applications [10].

Global interest in cellulose-based materials has grown significantly in recent years. Researchers continue to conduct extensive scientific studies on their properties, while practical applications expand across a wide range of fields. Cellulose-based nanostructured photocatalyst hybrids have gained particular attention in recent years, and the number of studies in this area continues to rise steadily.

CNCs possess several advantageous properties, including distinctive optical features, high stability, large surface area, and excellent mechanical strength, with a tensile capacity of about 10 GPa and a Young’s modulus of 140–150 GPa [11]. While CNFs can hold vast amounts of water, frequently exhibiting swelling ratios of 100 to 1,000 grams of water per gram of dry cellulose, which is 10,000% to 100,000% swelling capacity, while maintaining their structural integrity [12].

**Figure 2:**
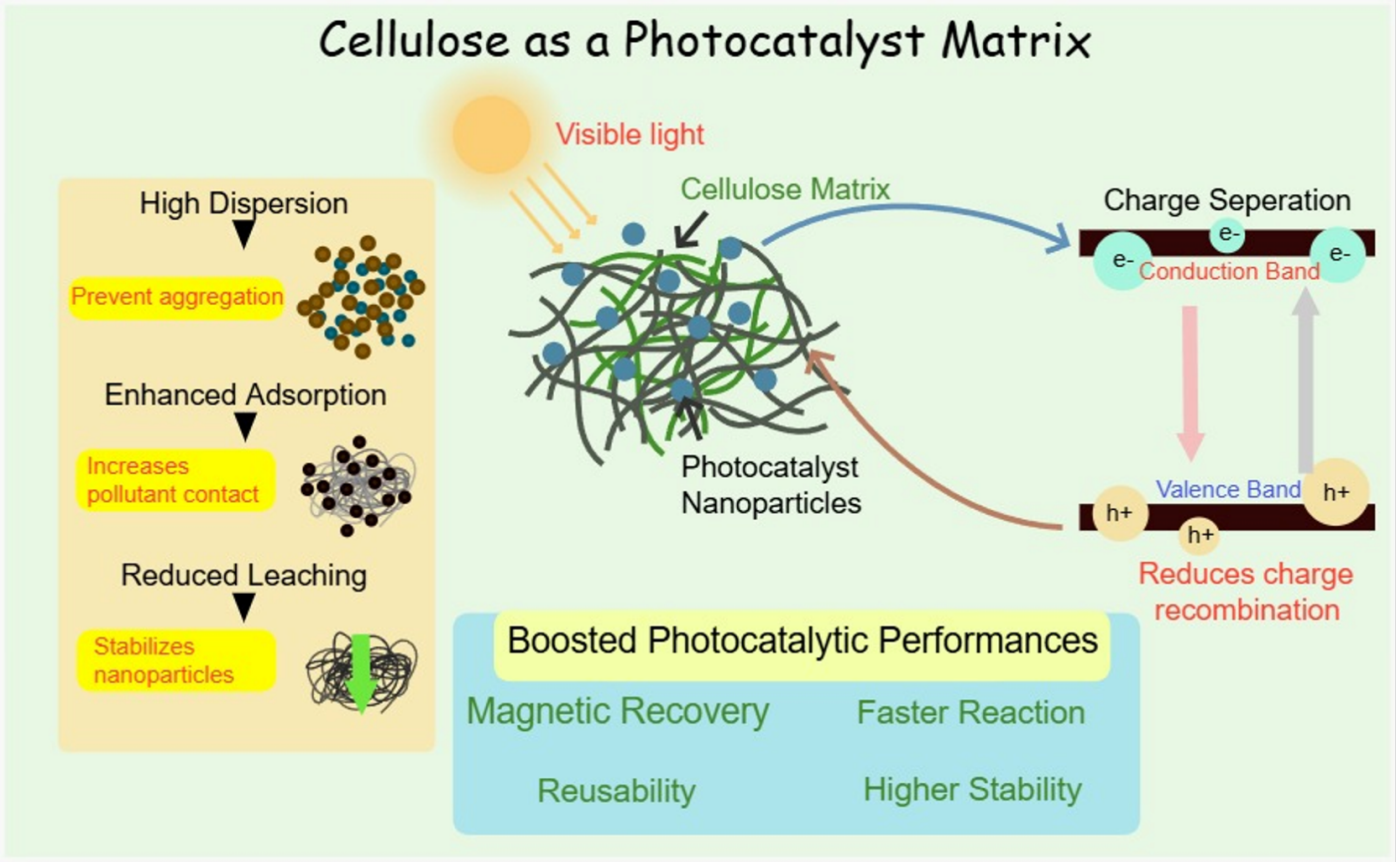
Illustration of the role of cellulose as a photocatalyst matrix. Diagram created with Chemix (https://chemix.org).

Cellulose possesses strong mechanical properties, and its favorable dimensions and strength make it an ideal material for water treatment membranes. This led researchers to apply it as a base material for developing such membranes [13]. A new approach involves immobilizing photocatalysts on membrane surfaces to create a polymer photocatalytic membrane matrix, which has shown strong effectiveness in degrading water contaminants [14]. They also prevent biofouling, a major problem in membrane separation. Scientists are increasingly combining cellulose with different photoactive nanomaterials, including metal oxides and nonmetallic semiconductors, to remediate and purify water, and the use of these hybrid systems is growing steadily in water treatment applications [15]. Photocatalyst immobilization on membranes addresses key challenges by preventing photocatalyst loss through leaching while simultaneously expanding the range of practical applications. These combined benefits make photocatalytic polymer membranes highly promising, as they enhance both membrane filtration and photocatalytic degradation, creating a dual-action approach that significantly improves overall water treatment performance as shown in Figure 2 [14].

**Figure 3:**
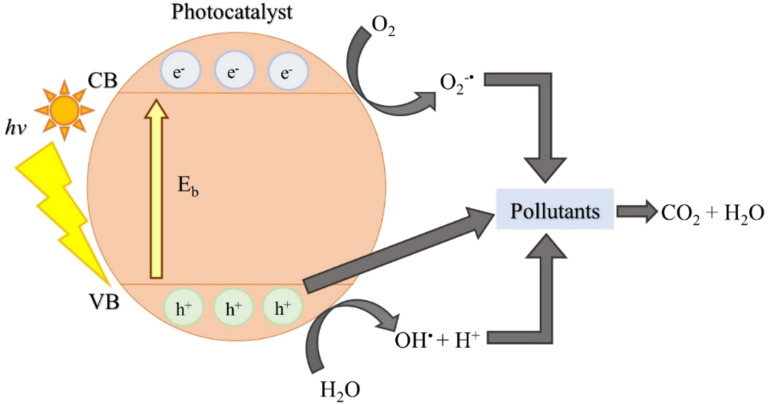
Mechanism of photodegradation of organic pollutants by photocatalyst.

Photocatalytic degradation is an advanced oxidation process (AOP) in which a catalyst is used to accelerate the degradation or breakdown of organic pollutants in water or air using light energy. This AOP relies on the ability of the catalyst to absorb photons from light, typically ultraviolet (UV) radiation or visible light, and generate reactive oxygen species (ROS) such as hydroxyl radicals (^•^OH) or superoxide radicals (O_2_^•−^) through photoexcitation [16–17]. In photocatalytic degradation, the catalysts typically used are semiconductor materials that can absorb light energy and generate electron–hole pairs, which then participate in redox reactions to produce ROS that degrade organic pollutants. Titanium dioxide (TiO_2_) and zinc oxide (ZnO) are the common catalysts that have been used [18–19]. Figure 3 illustrates the general mechanism of photodegradation of organic pollutants by photocatalyst.

This review aims to summarize current methods for extracting cellulose-based materials, focusing on their technical, economic, and environmental aspects. Key considerations include yield, purity, energy use, and scalability. The discussion includes mechanical, chemical, and biological approaches. It also discusses methods for immobilizing photocatalysts in cellulose membranes. By comparing these methods, this work provides insights into selecting optimal extraction techniques for specific applications.

## Review

### Cellulose and nanocellulose

Cellulose is a natural biopolymer composed of glucose units arranged as a water-insoluble polysaccharide with a high molecular weight, and it typically makes up 40–60% of plant cell walls. The remaining components of the cell wall include hemicellulose (15–30%) and lignin (2–35%), which form a cross-linked matrix around the cellulose fibers and bind covalently to the crystalline cellulose structure [20]. Its polymerization degree ranges from 10,000 to 20,000 units, depending on the source material. Every glucose monomer in cellulose contains three hydroxy groups that determine its crystalline structure and physical characteristics as these groups form hydrogen bonds with neighboring oxygen atoms to stabilize the glucose connections and create straight cellulose chains. In addition to shaping the structural arrangement, the hydroxy groups also control cellulose’s chemical behavior since their reactive sites allow for chemical modifications that enable diverse functionalization of cellulose materials [21].

Cellulose is available in several commercial forms, including microfibrillated cellulose (MFC) and microcrystalline cellulose (MCC); other important variants include CNCs, microfibrils, and bacterial cellulose (BC). Manufacturers produce cellulose at both the microscale and the nanoscale, where materials such as MCC consist of microscopic particles up to 1 µm in length and over 1 µm in width; MFC is typically obtained through mechanical or chemical treatment of wood, and microfibrils appear as individual fibers measuring more than 10 µm long and 2–20 µm wide [22]. At the nanoscale, cellulose is transformed into nanocellulose, which includes CNCs produced by acid hydrolysis and typically measuring 50–500 nm in length and 2–20 nm in width, as well as BC, which forms through bacterial synthesis and shows high crystallinity, excellent elasticity, and mechanical strength, with fibers ranging from 200 to 3000 nm long and 10–75 nm wide [23]. Extraction at the nanoscale not only reduces structural defects within cellulose’s hierarchy but also introduces new characteristics such as increased surface area, thereby broadening its application potential [22].

CNCs are increasingly gaining attention as reinforcing materials because they are green, biodegradable nanoparticles with great potential for developing sustainable polymer composites, and their production relies on abundant natural raw materials that are available at low cost, making them economically attractive [24]. CNCs combine exceptional mechanical properties with both high strength and impressive flexibility, and their dynamic mechanical performance makes them particularly promising for advanced applications. In recent years, researchers have focused on extracting CNCs from a variety of low-cost and renewable waste sources, including cardamom, waste cotton cloth, and even marine biomass such as sargassum, which further strengthens their value as sustainable and eco-friendly materials [24–26].

### Sources of cellulose

Cellulose is one of the most abundant natural materials, and it exists in many different sources that can be used to create valuable products. These sources are generally divided into four main groups, which are plants, waste materials, bacteria, and marine organisms. Plants represent the largest and most important source of cellulose, with trees providing substantial amounts through both softwood species like pine [27] and hardwood species such as birch [28]. Among plant-based sources, cotton fiber stands out as a key material for textiles as it is not only renewable and widely available but also cost-effective, highly biocompatible, and fully biodegradable, which makes it particularly attractive for diverse applications [29]. In addition to these conventional sources, agricultural residues such as rice straw have gained attention as sustainable alternatives, since they are renewable resources that help reduce waste while avoiding the need for cutting down trees [30].

Another valuable source of cellulose comes from different types of waste such as old newspapers, office paper, and cardboard boxes, which can be recycled into cellulose through proper treatment and extraction steps [31]. Beyond paper waste, a wide range of food residues including banana peels [32], garlic skins [33], and various nut shells [34], also provide useful amounts of cellulose, turning everyday organic waste into a renewable raw material for sustainable applications.

A third important source is bacterial cellulose, which is produced by tiny living organisms during fermentation in sugar-rich liquids such as glycerol [35–36]. This form of cellulose is highly pure and composed of extremely small and thin fibers, giving it unique qualities that make it valuable for a wide range of applications in sectors such as biomedicine, diagnostics, cosmetics, and water treatment [37]. Despite its many advantages, bacterial cellulose remains more costly to produce compared to cellulose derived from plants, which limits its large-scale use.

A fourth source of cellulose comes from certain sea animals such as tunicates, which produce cellulose in their outer coverings. The nanocrystals extracted from tunicates have distinct advantages over other varieties, as they exhibit much higher aspect ratios than plant-derived cellulose nanocrystals and possess a Young’s modulus that even surpasses that of bacterial cellulose nanocrystals [38–39]. These exceptional properties make tunicate cellulose particularly valuable for advanced and specialized applications. However, because these sea creatures are small and difficult to collect, this source remains uncommon and is mainly limited to research purposes.

### Extraction and isolation of nanocellulose

Recently, researchers have extracted and isolated CNCs using a variety of methods, including acid hydrolysis [3,24], enzymatic hydrolysis [8,40], TEMPO-mediated oxidation [41–42], ionic liquids [10,20], steam explosion [25], as well as combined approaches that integrate multiple techniques [24–25,41]. Each of these extraction strategies offers distinct advantages, such as preserving crystallinity, enhancing surface functionality, or reducing chemical waste; yet, each method also presents certain limitations, including environmental concerns, high cost, or technical complexity. Table 1 summarize the cellulose yield, nanocellulose yield, and crystallinity index from different extraction methods.

**Table 1:** Cellulose yield, nanocellulose yield, and crystallinity index from different extraction methods.

Extraction method	Cellulose yield	Nanocellulose yield	Crystallinity index	References

acid hydrolysis	N/A	40–90%+	70–90%+	[3,41,43–45]
enzymatic hydrolysis	N/A	75–90%	75–85%	[8,40,46–48]
TEMPO-mediated oxidation	N/A	80–95%	65–80%	[41–42,49–51]
ionic liquids	≈65%	60–75%	50–80%	[10,20,44,52–53]
steam explosion	40–75%	low	60–75%	[54–58]

**Acid hydrolysis.** Acid hydrolysis remains the most widely used method for extracting CNCs; researchers typically treat cellulose fibers with strong mineral acids such as sulfuric acid, phosphoric acid, or hydrochloric acid [44]. Before hydrolysis, the raw materials usually undergo several pre-treatment steps, including washing, drying, dewaxing, alkali treatment, and bleaching, to remove impurities and enhance accessibility. During hydrolysis, the acid selectively breaks down the amorphous regions of cellulose while preserving the crystalline domains, producing highly ordered nanocrystals. The reaction is usually carried out within a temperature range of 45–65 °C for periods ranging from 15 min to 10 h at lower temperature, resulting in CNCs that are highly pure and well-dispersed in water [3,8,24,41–42,59].

Acid hydrolysis is a kinetically driven process. At room temperature, the reaction is far too slow, taking more time to achieve high yields. Increasing the temperature provides the necessary activation energy to speed up the reaction. However, temperatures higher than 65 °C will trigger unwanted side reactions, such as dehydration of glucose molecules, and lower the yield. Time directly influences the physical dimensions of the resulting CNCs. If the reaction is stopped too early, the fibers remain entangled and too long. If left for too long at a relatively high temperature, the acid will start to depolymerize cellulose into simple sugars.

**Figure 4:**
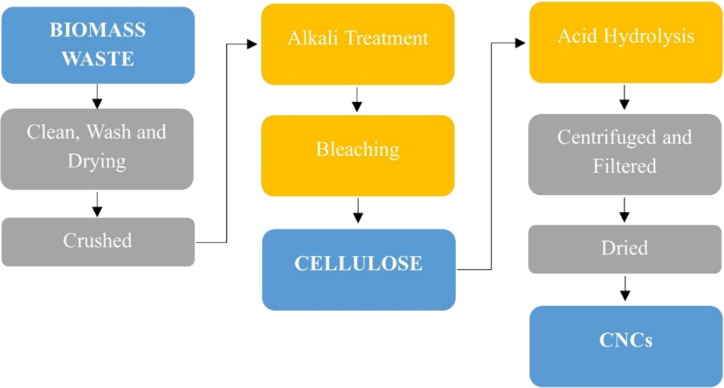
Schematic diagram of cellulose nanocrystals preparation process.

However, this method generates large amounts of acidic waste that must be neutralized, raising environmental concerns and complicating wastewater management. Sulfuric acid poses significant limitations, as its strong oxidative nature often causes cellulose degradation during hydrolysis, leading to relatively low CNC yields [24]. To address these challenges, researchers have explored alternative acids, such as hydrochloric acid, which produces CNCs with weaker oxidation effects and minimal thermal degradation, though it faces difficulties in maintaining stable dispersions. Recent studies have also examined mixed-acid systems, which aim to balance reactivity and dispersion by blending different mineral acids. Yet, while this approach appears promising, further investigation is still needed to optimize processing conditions and improve scalability [41,59]. Figure 4 shows the schematic diagram of cellulose nanocrystals preparation process.

**Enzymatic hydrolysis.** Enzymatic hydrolysis provides an environmentally friendly approach to producing CNCs as it breaks down cellulose fibers under mild conditions that typically involve temperatures of 36–50 °C and a neutral pH, with reaction times ranging from 24 to 72 h, making the process both safer and more sustainable compared to conventional chemical treatments [8,40,59]. Comparable to acid hydrolysis, setting the temperature to an optimum range can effectively increase the amount of CNC yield. A suitable reaction time can also ensure quantity and quality of the extracted CNCs. This method requires only minimal amounts of water, produces no toxic byproducts, and even allows for the recovery of useful sugar byproducts, which further enhances its sustainability. CNCs obtained through enzymatic hydrolysis exhibit several unique advantages, including improved thermal stability, more uniform particle sizes, and higher crystallinity relative to those produced by acid hydrolysis [8,46]. Despite these benefits, the process is generally slower and more costly, largely due to the high price of enzymes. Cellulases act as the primary enzymes in this method, although other enzymes may also participate in breaking down fibers, contributing to the overall efficiency of the process [40].

**TEMPO-mediated oxidation.** 2,2,6,6-Tetramethyl-1-piperidinyloxy (TEMPO)-mediated oxidation is a widely studied chemical method for producing CNCs. TEMPO acts as the catalyst, and the process is followed by mechanical disintegration to obtain nanoscale structures [41,60]. This technique generally produces nanofibers with nanoscale diameters and microscale lengths, making them particularly suitable for advanced applications. The reaction involves TEMPO in combination with sodium hypochlorite (NaClO) as the oxidant; during the process, TEMPO selectively oxidizes cellulose surfaces by introducing carboxyl groups. These negatively charged groups enhance electrostatic repulsion between fibers, which helps to separate individual microfibrils and effectively disintegrates cellulose into nanoscale components [41]. Despite its effectiveness, TEMPO-mediated oxidation has notable drawbacks, such as reliance on toxic chemicals, multistep procedures that increase complexity, and limitations caused by the restricted number of accessible oxidation sites on cellulose [60].

TEMPO oxidation is a highly sensitive catalytic cycle. The parameters must be tightly controlled to ensure that the reaction only modifies the surface groups without depolymerization. It is usually carried out at room temperature with a pH around 10 and low doses of TEMPO (0.1 mmol) [61]. The oxidation reaction actively consumes base and produces acid, causing the pH to drop continuously. It must be maintained by adding NaOH dropwise. This is because NaClO is most active at pH 10, and the newly formed carboxyl groups are ionized into carboxylates, driving the reaction forward [61]. The reaction should be maintained at room temperature to prevent degradation of TEMPO radical. TEMPO acts as a catalyst, and it will continuously regenerate during the reaction. It is also expensive and mildly toxic, so keeping its concentration as low as possible is vital for economic and environmental reasons.

**Ionic liquids.** Recent studies have highlighted ionic liquids (ILs) as highly promising solvents for lignocellulosic biomass pretreatment, since these salts remain liquid at relatively low temperatures and are valued for their environmental advantages and energy-efficient processing conditions [62]. Unlike conventional solvents, ILs are non-flammable, thermally stable, and exhibit negligible vapor pressure, which not only minimizes safety hazards but also reduces volatile emissions, making them attractive for sustainable applications. Their effectiveness arises from unique molecular interactions, as a wide range of ILs demonstrate strong biomass-dissolving abilities primarily through hydrogen bonding and electrostatic forces. During pretreatment, the anions of ILs first interact with cellulose hydroxy groups, causing the biomass to swell, while the bulkier cations subsequently surround individual cellulose chains to form solvation cages that drive the fibers apart through electrostatic repulsion [10,20]. This two-step mechanism allows for efficient cellulose extraction while maintaining structural integrity; however, the processing temperature plays a critical role. Higher temperatures can accelerate pretreatment, but there is also a risk to degrade valuable biomass components, whereas lower temperatures preserve these components but require impractically long processing times for industrial use [10].

**Steam explosion.** Steam explosion is an effective technique for isolating nanofibers from biomass. It operates through two main steps involving high-pressure steaming followed by rapid pressure release [54–55]. In the first stage, dry biomass absorbs steam under elevated pressure and temperature, and when the pressure is suddenly released, the rapid drop causes instant water evaporation, generating strong forces that rupture and break apart the material. This approach not only isolates nanofibers but also serves as an efficient pretreatment, preparing the biomass for subsequent processes that refine it into nanoscale structures. Mechanical methods are often employed after steam explosion, with ultrasonication proving particularly effective, as it uses high-frequency sound waves to disrupt fiber structures, removing non-cellulosic substances that normally bind the fibers together. Once these cementing materials are broken down, individual nanofibrils separate from larger fiber bundles, and the resulting nanofibrils display improved performance in composites, where their enhanced dispersion significantly strengthens reinforcement properties.

**Combined methods.** Many researchers are now combining different techniques such as applying acid hydrolysis pretreatment followed by oxidation [41] or using mild acid hydrolysis in combination with steam explosion [25]. These integrated approaches help balance speed, cost, and environmental impact while at the same time improving the overall quality and performance of the extracted CNCs.

**Comparison among methods.** To fully assess these extraction methods, we must transition from a small-scale synthesis to commercial manufacturing that requires balancing yield and particle quality against environmental impact, reagent costs, and energy consumption. Despite its significant environmental drawbacks, sulfuric acid hydrolysis remains the major extraction method for the large-scale production of CNCs. It produces highly crystalline CNCs with excellent colloidal stability. However, it generates massive volumes of highly corrosive, toxic, and acidic wastewater. The cost of neutralizing the acidic wastewater and recovering the acid limits broader expansion, especially in regions with strict environmental regulations. For the production of CNFs, TEMPO oxidation is currently the most promising and successfully commercialized chemical route. Reagents such as TEMPO and NaClO are expensive and toxic, and the operating process requires precise, continuous pH monitoring [61]. Looking forward, purely chemical methods are unsustainable. Combined methods represent the most economically and environmentally promising frontier for future large-scale production. By using a highly scalable physical process like steam explosion to process agricultural waste, the processed biomass can be hydrolyzed effectively by mild, recoverable organic acids or low doses of enzymes. This lowers the chemical footprint drastically, eliminates toxic wastewater, and enables inexpensive feedstocks, addressing the core limitations of purely chemical methods. Table 2 shows the operation steps, advantages, and disadvantages of each extraction/isolation method.

**Table 2:** Operation steps, advantages, and disadvantages of each extraction/isolation method.

Extraction/isolation method	Operation steps	Advantages	Disadvantages

acid hydrolysis	Cellulose fibers were treated with strong acids to break down the less organized parts of the cellulose, leaving behind the crystalline nanocrystals.	It produces highly crystalline CNCs with uniform sizes. The process is relatively fast and works well with various cellulose sources.	The method generates acidic waste, requiring neutralization and disposal. The strong acid can also degrade cellulose if not carefully controlled.
enzymatic hydrolysis	A greener approach, in which enzymes are used instead of harsh chemicals. Cellulase enzymes naturally break down cellulose into smaller parts.	Enzymes offer an eco-friendly alternative. They work under mild conditions and preserve crystallinity. It also produces fewer harmful byproducts.	Enzymes are expensive and the reaction is slower than acid hydrolysis. The process may also need pretreatment to improve efficiency, increasing the complexity.
TEMPO-mediated oxidation	This technique uses TEMPO to add negatively charged groups to the CNCs, helping them disperse better in water; it is followed by mechanical disintegration to yield nanoscale cellulose.	This method modifies the surface of CNCs with carboxylate, enhancing water solubility and stability in composites. It works under mild conditions and yields uniform nanoparticles.	TEMPO and other chemicals used are costly, and the reaction requires precise pH and temperature control. Oxidation may also reduce CNC yield by breaking down crystalline regions.
ionic liquids	Ionic liquids are liquid salts that dissolve cellulose at low temperatures. After dissolving, water will be added to reform the cellulose as tiny crystals.	Ionic liquids dissolve cellulose at low temperatures and can be recycled, thus reducing waste. They work with diverse feedstocks. The process also preserves cellulose integrity well.	The liquids are expensive, and some types are toxic or hard to purify for reuse. Scaling up the process is challenging due to high costs and solvent recovery requirements.
steam explosion	The plant fibers are cooked with steam under high pressure, which is suddenly released. The pressure changes burst the fibers into smaller parts.	This method uses no chemicals, making it eco-friendly. It efficiently breaks down raw biomass and can be combined with other methods for better results.	The CNCs may contain impurities, and additional purification may be required. The process also requires high energy for steam generation and specialized equipment.
combined methods	Mixing different techniques	Hybrid approaches which balance efficiency and sustainability. They reduce chemical use, energy consumption, and waste while improving CNCs quality.	Combining methods increases process complexity and cost. Optimizing conditions for each step can be time-consuming.

### Uses of extracted cellulose

As the world’s economy continuously depends heavily on fossil fuels, the need to reduce carbon emissions has pushed renewable biomass into the spotlight as a promising alternative for producing biofuels and bio-based products, with cellulose, which is a natural, renewable, and biodegradable polymer, emerging as a central material in this transition. The modification and functionalization of CNCs open exciting opportunities for creating advanced materials with enhanced or entirely new properties; the incorporation of specific chemical groups or materials enables synergistic effects that can impart electronic, magnetic, catalytic, fluorescent, or optical functionalities, thereby improving performance and expanding applications into more specialized fields. Today, cellulose and its derivatives are already being used across diverse sectors including packaging, construction, and electronics [63–64]. Research continues to focus on extracting cellulose primarily from plants such as wood, cotton, and agricultural residues and processing it through various nanotechnology-based methods to enhance its mechanical and thermal properties, ultimately allowing this abundant biopolymer to play an increasingly important role in both established and emerging applications [65].

In the papermaking industry, cellulose fibers derived from wood pulp or other plant sources form the essential raw material, and these fibers interlock through hydrogen bonding to impart both mechanical strength and flexibility to the paper matrix, enabling the production of a wide range of paper products with tailored properties [64]. The fiber morphology and the degree of polymerization of cellulose strongly influence the quality and performance of the final product, making cellulose a critical factor in determining paper characteristics. More recently, bacterial cellulose has emerged as a natural material of particular interest due to its eco-friendly production process, low cost, strong water absorption, high biocompatibility, and biodegradability, which collectively make it a valuable resource across different applications. In papermaking, bacterial cellulose has been incorporated as a high-strength additive to create fine fiber networks, while also being explored for restoring damaged paper and even in the development of magnetic paper designed for anti-counterfeiting purposes [66].

Cellulose has a long history of use in the biomedical field. Today, several forms of medical-grade cellulose are widely available, including propellant cellulose, MCC, and its common derivatives such as carboxymethyl cellulose and methyl cellulose [67]. The medical sector benefits greatly from cellulose’s natural biocompatibility, while its derivatives and nanocellulose can be produced through chemical modifications or combined physical and chemical treatments, which have further expanded its applications by offering better solubility, improved compatibility with biological systems, and the ability to be tuned or functionalized for the development of advanced composite materials [68–69]. These forms of cellulose are particularly valuable in producing highly purified wound dressings that promote tissue regeneration, especially for burns and chronic wounds, highlighting their crucial role in modern biomedicine. A notable example is cellulose acetate (CA), a natural and environmentally friendly derivative obtained from renewable sources such as wood pulp, which combines non-toxicity with water-attracting properties, robust chemical and mechanical strength, and relatively high heat resistance. These features make CA an essential material for diverse biomedical applications, including drug delivery systems, wound care products, and scaffolds for tissue engineering [70–72], reinforcing its position as a reliable and sustainable choice for next-generation healthcare solutions.

In recent years, cellulose-based fibers, films, and aerogels have found widespread application in drug delivery, where cellulose typically serves as the foundational material that can be further modified to enhance its performance. These modifications not only increase the drug loading capacity and extend the release time of therapeutic agents but also contribute to improved antimicrobial activity, biocompatibility, and mechanical strength, making cellulose a versatile platform for advanced delivery systems [69]. Depending on the processing method, the derived cellulose may exist in both amorphous and crystalline forms with various structural variations such as macrofibers, fibrils, pores, and both micro- and nanocrystals, each offering distinct advantages for medical applications. Within drug delivery research, nanoscale systems have gained prominence because of their ability to selectively target specific organs and tissues, thereby reducing side effects, minimizing systemic toxicity, and improving overall treatment efficiency [23].

Researchers are increasingly developing cellulose-based scaffolds for tissue engineering as these structures can mimic the human extracellular matrix and show strong potential for applications in cartilage and bone regeneration. Among different cellulose sources, BC has gained particular attention because it combines strong mechanical properties with controlled biodegradability and natural biocompatibility, while its microbial origin ensures a high degree of purity that makes it especially suitable for biomedical use [73]. Calcium phosphate cements (CPCs) are valuable materials in bone-related applications due to their intrinsic bioactivity and their ability to form hydroxyapatite, the mineral that closely resembles the inorganic component of bone. When BC is incorporated into CPCs, it further enhances their compressive strength without diminishing their capacity to support bone growth or to be safely resorbed by the body [73–74]. Beyond regenerative medicine, researchers have also designed hybrid biosensors using cellulose nanocrystals combined with magnetite to create (x-CNC)-Fe_3_O_4_ systems, which demonstrate excellent biocompatibility and are tailored for glucose detection. These biosensors are often fabricated as thin-film strips that can be applied directly onto the skin or tongue, where they detect glucose levels non-invasively through sweat or saliva, offering a promising platform for biomedical monitoring [75].

Hydrogels are highly water-absorbing materials with a three-dimensional network structure, and they are widely recognized for their flexibility, responsiveness to external stimuli, tunable performance, and strong biocompatibility, which collectively make them an excellent base material for wearable electronic devices [76–77]. Traditional hydrogels mainly contain water; water molecules form extensive hydrogen bonds that unfortunately lower the material’s resistance to cold conditions. As the temperature falls below 0 °C, the hydrogel begins to freeze, leading to a loss of flexibility, reduced stretchability, and eventual failure of its functional properties [78]. To overcome this critical limitation, researchers have introduced nanocellulose into hydrogel systems, creating nanocellulose-based hydrogels that provide enhanced stability and maintain performance even under challenging temperature conditions [77–78].

MCC bio-composites are emerging as a practical and sustainable alternative to conventional polymer-based composites, offering eco-friendly solutions that can address pressing environmental concerns while supporting the development of innovative products for modern applications. Researchers are increasingly focusing on MCC-reinforced bio-composites to meet the rising demand for sustainable, high-performance, and cost-effective materials, with the aim of creating engineered composites that combine strong durability with full environmental sustainability [79]. The growing interest in isolating and utilizing MCC fibers in composite materials arises from their remarkable mechanical strength, stiffness, and lightweight characteristics, as well as their renewable and biodegradable nature.

Nanocellulose is widely regarded as an effective reinforcing filler because its tiny structures possess a high aspect ratio, excellent mechanical strength, and a large number of hydroxy groups that not only enable extensive hydrogen bonding but also allow for chemical modifications, making it highly compatible with a broad range of polymer materials. Among the many approaches for chemical modification, TEMPO-mediated oxidation is one of the most common, as it selectively converts the primary hydroxy groups at the C6 position of cellulose into carboxylate groups under mild reaction conditions, thereby improving the colloidal stability of the nanofibers. Through this modification, nanocellulose gains the ability to form stronger interactions with hydrogels and composite materials, which further enhances its reinforcing performance and widens its potential applications [80–81].

CNCs have become a central focus in advancing membrane technology for water treatment as conventional membranes, though vital for protecting aquatic life, often struggle to meet the rising environmental and social demands; one effective strategy to overcome these limitations is through membrane modification [82]. In this context, nanocellulose has emerged as a promising renewable nanomaterial because it is a sustainable, carbon-neutral biopolymer that combines wide availability, low cost, excellent flexibility, large surface area, ease of modification, and high biocompatibility, making it particularly attractive for water purification applications. Owing to these features, CNCs have been widely studied not only as adsorbents, supports for catalysts, and coagulants or flocculants, but also as membrane materials, with recent research showing that CNCs can either be incorporated into existing membranes or used to develop entirely new high-performance ones [83]. The effectiveness of CNCs is due to especially the high surface area and the abundance of hydroxy groups, which enable improved interaction with water contaminants while also aligning with environmental sustainability goals [82–83]. In practical applications, the addition of CNCs has been shown to enhance several key membrane characteristics, including increased hydrophilicity, greater mechanical strength, and reduced susceptibility to irreversible fouling. These combined improvements result in CNCs-based membranes that are not only more effective but also more reliable and durable for long-term environmental applications [82].

Substrates form the foundation of optoelectronic devices and play a decisive role in determining their overall performance, since properties such as mechanical strength, optical transparency, and maximum processing temperature directly influence whether a substrate is suitable for applications. Traditionally, glass and plastic substrates have dominated the optoelectronic industry, especially in the development of flexible electronics, yet recent research has highlighted transparent nano-paper derived from renewable cellulose nanofibers as a highly promising alternative. Unlike plastic, this nano-paper is more environmentally sustainable because it originates from natural materials, while its unique fibrous nanocellulose structure not only reduces environmental impact but also introduces new functionalities that can significantly enhance the performance of electronic and optoelectronic devices [84].

Researchers have recently developed CNC network structures as versatile substrates for catalytic reactions, including reduction, oxidation, and coupling processes, and one of their key advantages lies in the ability to form porous networks with reactive surfaces that not only store inorganic nanoparticles (INPs) but also facilitate their easy diffusion throughout the structure. This unique design enables the creation of CNC–INP networks with exceptionally large surface areas, making them highly effective for catalytic applications [67]. At the same time, advances in synthetic methods now allow for precise control over the size, shape, and aspect ratio of metal nanomaterials, parameters that directly determine their catalytic properties. Yet, a major challenge persists because INPs are kinetically unstable and their production often requires large amounts of solvents, reagents, reducing agents, and capping agents to ensure stability [67,85].

In recent years, metal–organic frameworks have attracted considerable attention as versatile supports or precursors for catalysts because of their high porosity, tunable functions, customizable structures, and abundance of potential anchoring sites, making them particularly valuable in advanced catalytic applications. Among these, photocatalysis has emerged as one of the most rapidly developing AOPs, in which light activates a catalyst and triggers chemical reactions that are widely applied in wastewater treatment to degrade organic pollutants efficiently [86]. Metal oxides serve as key activators in photocatalytic oxidation by initiating radical chain reactions, and they remain favored for their low cost, minimal toxicity, and ease of modification through strategies such as size reduction and doping, which enhance their reactivity and overall performance [86]. Widely used examples include ZnO, TiO_2_, and iron oxide, which support a broad range of catalytic applications, and researchers have explored several production methods for these nanoparticles, with the sol–gel process relying on significant amounts of alcohols and the hydrolysis–calcination method, which has already been commercialized but requires high energy input and the use of hydrochloric acid as a solvent [86].

CNCs possess several valuable features that make them excellent candidates for conductive nanocomposites as their nanoscale size and high aspect ratio help reduce the percolation threshold, while the abundance of surface hydroxy groups enables easy chemical modification to tune electrical properties [87–88]. Their strong mechanical strength supports the fabrication of self-standing materials without the need for additional additives, and their high carbon content and small size make them particularly suitable for producing porous carbon-based electrodes through high-temperature pyrolysis. Beyond enhancing conductivity, the use of CNCs as template materials provides multiple advantages, since they not only improve the strength of the resulting composites but also allow for water-based processing thanks to their excellent dispersibility in water and the presence of reactive hydroxy groups on their surface. To achieve conductivity, four main strategies are commonly applied, which are coating CNCs with conductive polymers, depositing metallic layers on them, converting them through carbonization, and integrating them with carbon nanotubes (CNTs), each approach offering unique pathways to tailor the performance of CNCs-based conductive nanomaterials [87].

### Cellulose-based nanostructured photocatalysts

Cellulose is the bulk, hierarchical material found in nature, containing a mix of crystalline and amorphous regions. CNCs are isolated by removing the amorphous regions, leaving only the highly ordered nanoscale crystals. Both prevent the aggregation of photoactive nanoparticles, keeping them dispersed so their active sites remain available [5]. Bulk cellulose functions primarily as a macroscopic structural support focused on easy handling, filtration, and recovery. CNCs function as a nanoscale active platform focused on maximizing surface area, preventing nano-catalyst aggregation, and actively enhancing charge-transfer dynamics [89].

Recently, nanocellulose has emerged as a promising support material for the synthesis of metal, metal oxide, and non-metallic nanoparticles, and when these components are combined, they often act synergistically to enhance photodegradation processes. Noble and transition metal nanoparticles such as iron, silver, palladium, platinum, titanium, and copper have been widely employed for breaking down pollutants; however, their tendency to aggregate limits performance, which is why they are frequently immobilized on solid substrates. This immobilization not only prevents clumping but also improves stability and allows nanoparticles to be reused in repeated experiments, making the overall process more efficient and sustainable [86,90].

Photocatalysis has become a cost-effective and attractive method for environmentally friendly applications, particularly in breaking down organic dyes in wastewater, producing hydrogen, and supporting antibacterial treatments. Its success largely depends on the properties of the semiconductor photocatalyst. An ideal photocatalyst should remain stable against photocorrosion, operate efficiently under a broad range of light wavelengths, and at the same time be affordable and non-toxic. Visible-light photocatalysts stand out as especially useful since they can absorb a large portion of solar energy, thereby increasing efficiency in practical applications [91]. Within this context, nanocellulose plays an important role in enhancing photocatalytic performance by promoting charge separation, supporting surface functionalization, and improving light absorption, while one of its most valuable contributions lies in reducing the recombination of photogenerated electron–hole pairs. Its large surface area further helps stabilize photocatalytic nanoparticles, creating favorable conditions for efficient charge transfer and overall reaction performance [90].

In photocatalysis, raw photocatalysts are typically fine nanopowders. They are excellent at using light to degrade pollutants, but they suffer from three major functional flaws. They tend to clump together to reduce their surface energy, thus reducing active surface area, they are difficult to filter out of water after use, and their photogenerated charges often recombine before they can do any useful work [92]. For a photocatalyst to work, light must excite an electron, leaving behind a positively charged hole, the oxygen-rich functional groups on the cellulose surface can interact with the electronic bands of the attached semiconductors and act as an electron mediator or sink [93]. The complex, porous internal architecture of a cellulose matrix also creates a “light-trapping” effect, which enhances the photocatalytic efficiency [94]. After the photodegradation process, the cellulose–photocatalyst composite can be simply removed from the reactor with tweezers or recovered via basic filtration, washed, and reused for multiple cycles without losing its catalyst performance [95]. Cellulose functions as a highly active structural matrix that directly solves these physical and chemical bottlenecks. Rather than just being a passive container, cellulose actively participates in the photocatalytic process [96].

TiO_2_ is one of the most widely studied semiconductor photocatalysts and is commonly applied in both photocatalysis and energy storage due to its affordability, non-toxicity, strong activity, and environmentally friendly nature [97]. Despite these advantages, TiO_2_ faces notable limitations as its relatively wide bandgap of about 3.2 eV and rapid electron–hole recombination rate significantly reduces its effectiveness under visible light, which represents nearly 45% of the solar spectrum. To address these challenges, researchers have developed a variety of modification strategies and hybrid approaches aimed at enhancing the visible light absorption of TiO_2_ and suppressing electron–hole recombination, thereby improving its efficiency and expanding its potential for practical applications [98].

Metal sulfide semiconductors such as CdS, MoS_2_, ZnS, and CuS often encounter the problem of photocorrosion, a self-oxidation process that significantly reduces their stability during photocatalytic reactions and limits their practical applications. To overcome these drawbacks, researchers have focused on combining different photocatalysts with a straightforward yet effective strategy that balances the strengths of each material and improves overall performance [97]. For example, Song et al. developed TiO_2_/CdS heterojunctions with a porous structure, high crystallinity, and a reduced bandgap, features that contributed to enhanced catalytic degradation efficiency. When tested with methyl orange, the TiO_2_/CdS composite demonstrated strong stability, excellent photocatalytic activity, and reliable performance across multiple degradation cycles, highlighting its potential as a durable and efficient photocatalyst [99].

Another key challenge in using TiO_2_ for industrial wastewater treatment is the difficulty of catalyst recovery, and a practical solution to this problem is the immobilization of the photocatalyst on a larger support, which makes it easier to separate and reuse the material after the treatment process [100]. In response to these limitations, researchers have increasingly turned their attention to carbon/TiO_2_ composites, as they not only reduce the recombination of photogenerated electrons and holes but also enhance the adsorption of contaminants while preventing the undesirable aggregation of TiO_2_ particles, thereby improving both the stability and efficiency of the photocatalyst [101].

There is strong compatibility between TiO_2_ nanoparticles and cellulose chains, as the interaction occurs through covalent bonding that reinforces the polymer structure, making the cellulose chain more rigid and requiring greater energy to break. By using cellulose films or membranes as supports for TiO_2_ nanoparticles, researchers not only strengthen the structural stability of the composite but also solve a major challenge in photocatalytic treatment, which is the recovery of TiO_2_ from water after use, thereby making the process more efficient and easier to manage [102]. This strategy also reduces the risk of TiO_2_ nanoparticle contamination in treated water, an important factor in protecting human health. The growing interest in cellulose films as supports for TiO_2_ is further driven by cellulose’s natural advantages: it is biodegradable, widely available, cost-effective, and highly versatile, with a structure that can be easily modified to suit diverse applications including photocatalytic degradation of pollutants [19]. In addition, the optical properties of transparent cellulose-based thin films enhance photocatalytic efficiency as their ability to transmit UV or visible light ensures greater light penetration, which in turn improves electron distribution and transfer to the TiO_2_ surface, ultimately boosting photocatalytic activity and making pollutant removal more effective [103].

Hamad et al. explored the potential of natural biopolymers, particularly cellulose, as a low-cost and widely available carbon source, noting that, while cellulose is highly functional due to its extensive hydrogen bonding network, this same property makes it difficult to dissolve in most common solvents, which in turn creates unique opportunities for its use as a structural template. This insolubility and inherent stability make cellulose especially attractive as a support material for the nucleation and growth of inorganic phases such as TiO_2_ on its fibrous surface, allowing researchers to exploit its natural abundance and structural characteristics to develop sustainable photocatalytic composites [101].

Mohamad Azuwa Mohamed et al. prepared a regenerated cellulose (RC) thin film using recycled newspaper as the cellulose source, creating a sustainable and low-cost material designed for photocatalytic thin film applications. To evaluate its performance, they tested the photocatalytic activity of the RC/N-TiO_2_ composite by examining its ability to degrade methylene blue under both artificial UV and visible light. The results demonstrated that embedding nitrogen-doped TiO_2_ nanorods within the RC matrix significantly enhanced the physical and chemical properties of the composite film. Importantly, the study showed that this nanocomposite film could be applied directly in water treatment processes without leaving photocatalyst residues in the treated water, addressing one of the key challenges in practical photocatalytic applications. Moreover, the strong photocatalytic activity under both UV and visible light highlights its potential as an environmentally friendly material for portable water treatment technologies, particularly for the removal of organic pollutants [102].

**Figure 5:**
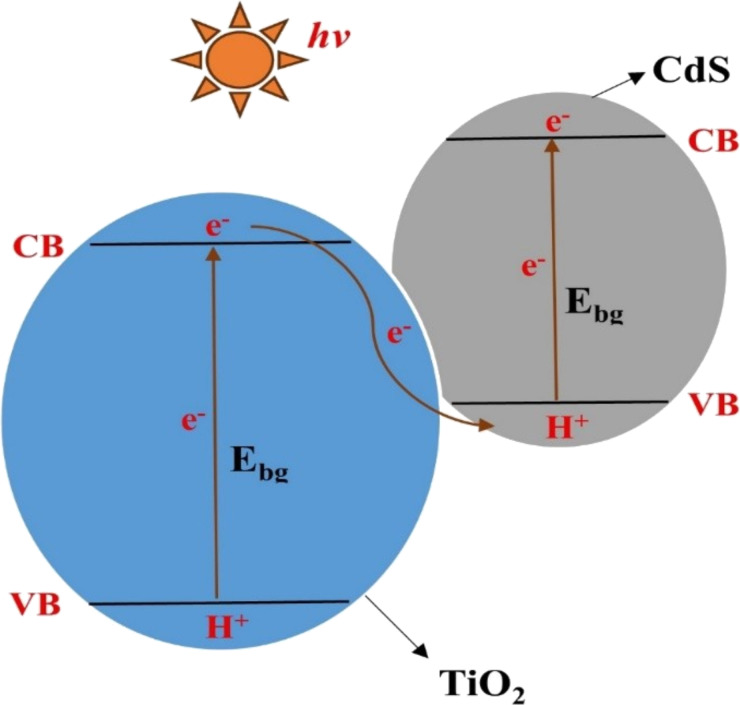
Potential positions of TiO_2_ and CdS band edges and schematic illustration of direct Z-scheme photocatalytic mechanism for BC@TiO_2_–CdS photocatalyst.

Qian et al. developed a photocatalytic gel by combining BC with TiO_2_–CdS nanocomposites using a microwave-assisted solvothermal method, creating a material specifically designed for removing methylene blue from water. This biomass-based photocatalyst successfully integrated the strong dye adsorption capacity of natural cellulose with the enhanced degradation ability of semiconductor nanomaterials, resulting in a dual mechanism that allowed for simultaneous adsorption and photocatalytic degradation, thereby increasing the overall methylene blue removal rate. A critical factor in this performance was the formation of a direct Z-scheme TiO_2_–CdS heterojunction, which effectively reduced electron–hole recombination and promoted efficient electron migration, leading to a significant improvement in photocatalytic efficiency [97]. Figure 5 shows the potential positions of TiO_2_ and CdS band edges and schematic illustration of direct Z-scheme photocatalytic mechanism for BC@TiO_2_–CdS photocatalyst.

ZnO nanoparticles can be effectively synthesized within a cellulose matrix, a strategy that not only prevents aggregation but also enhances dispersion stability, and Zheng et al. demonstrated this by successfully producing nanocrystalline ZnO particles through a simple in situ polyol method in which amidoximated bacterial cellulose (Am-BC) served as the template. The fine three-dimensional network of Am-BC, enriched with hydroxy and amidoxime groups, provided abundant reactive sites for ZnO nucleation and growth, effectively acting as a nanoreactor that improved both the yield and uniformity of ZnO nanoparticles compared to unmodified bacterial cellulose. This structural framework not only prevented clumping but also supported the assembly of well-distributed ZnO nanoparticles within the matrix; under optimized conditions, the resulting Am-BC/ZnO composites displayed excellent photocatalytic performance, highlighting the potential of functionalized cellulose supports for stabilizing semiconductor nanoparticles and enhancing their environmental applications [103].

Vu Hoai An et al. extracted cellulose from Nypa fruticans in Vietnam and converted it into CNCs through hydrolysis, before combining the CNCs with ZnO via a precipitation technique that relied on strong electrostatic interactions to ensure even dispersion of ZnO across the CNCs surface. This process yielded ZnO/CNCs nanohybrids with high thermal stability, where ZnO nanocrystals of about 50 nm were uniformly distributed due to the strong attraction between Zn^2+^ ions and the carboxyl groups present on the CNCs. Importantly, the study showed that methylene blue degradation was driven by a synergistic mechanism, combining the adsorption ability of CNCs with the photocatalytic activity of ZnO, which significantly enhanced overall removal efficiency; under optimized conditions, the system achieved up to 95% degradation of methylene blue using 40 mg of ZnO/CNCs in 15 mL of dye solution under a 15 W UVC lamp [104].

Leite et al. developed eco-friendly photocatalysts by immobilizing ZnO onto MFC through a simple, one-step in situ hydrothermal process carried out at low temperature, a practical method that requires only a few steps and is therefore suitable for large-scale applications. The resulting MFC@ZnO composites were tested for photocatalytic activity under UV-A, UV-C, and simulated solar light (SSL). In every case, they demonstrated outstanding performance, achieving more than 99% degradation of norfloxacin (NOR) within just 60 min. This remarkable efficiency highlights the composites’ ability to absorb and convert a wide spectrum of light into usable energy for pollutant breakdown, confirming their strong potential in real-world water treatment. Importantly, the integration of ZnO nanostructures with MFC not only enhanced photocatalytic performance but also advanced the development of greener, more sustainable materials; compared with other photocatalysts reported in the literature, these composites displayed superior efficiency, further underscoring their promise for sustainable environmental applications [105].

It is widely recognized that photocatalysts must not only exhibit high activity but also maintain strong stability throughout repeated use, and this was clearly demonstrated in the case of the ZnO/nanofibrillated cellulose (NFC) composite, whose durability was evaluated by performing five consecutive degradation cycles of methyl orange under identical UV irradiation conditions. After each cycle, the spent composite was carefully recovered by centrifugation, thoroughly washed with ethanol, and dried before being reused, the photocatalyst consistently retained both its activity and structural stability across all five runs. These results confirm that the ZnO/NFC composite holds great promise as an effective and reusable photocatalyst for dye degradation under UV light. Moreover, the unique structural characteristics of the composite, particularly its small fiber size and high aspect ratio, enabled the formation of a highly efficient network structure, which significantly enhanced photocatalytic performance. This optimized architecture proved remarkably effective, as the composite achieved complete (100%) degradation of methyl orange within 9 h of UV irradiation, further emphasizing its potential for practical wastewater treatment applications [106].

**Figure 6:**
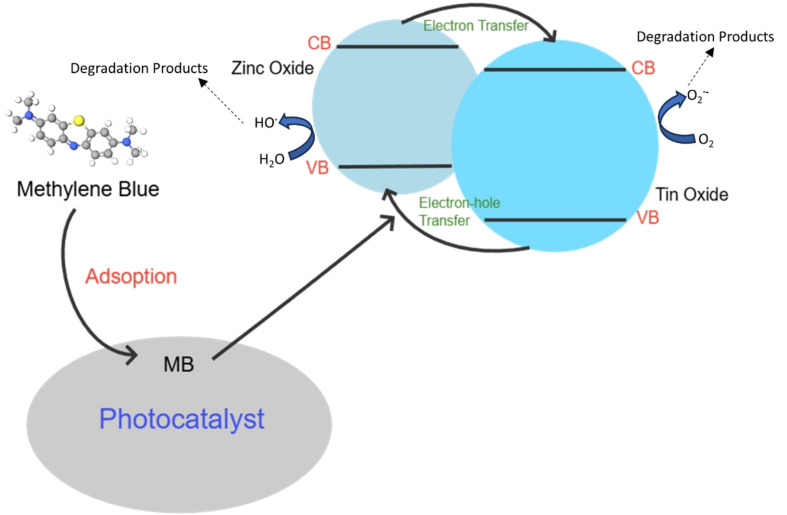
Schema of the simultaneous adsorption-photocatalysis process.

Perciani et al. explored the use of cross-linked cellulose beads as a sustainable and low-cost support for a ZnO/SnO_2_/carbon xerogel hybrid photocatalyst, with particular emphasis on its potential applications in effluent treatment. Their findings demonstrated that the presence of simulated sunlight greatly enhanced methylene blue removal across all test cycles, highlighting the effectiveness of this cellulose-supported system. In the first cycle, for example, the combination of adsorption and photocatalysis resulted in nearly a 40% increase in methylene blue removal, clearly showing that immobilizing the photocatalyst within the cellulose beads significantly improved photodegradation efficiency. The observed performance was largely attributed to the synergistic interaction between adsorption and photocatalysis, which allowed pollutants to be both captured and degraded more effectively. Furthermore, mechanistic insights obtained using the scavenger method revealed that the degradation pathway was dominated by the generation of reactive oxygen species, particularly hydroxyl and superoxide radicals, produced by the cellulose bead-supported catalyst under simulated sunlight [107]. Figure 6 shows a schema of the simultaneous adsorption-photocatalysis process.

Copper(I) oxide is a p-type semiconductor with a narrow bandgap of 1.8–2.5 eV, enabling it to respond effectively to a broad portion of the solar spectrum and making it an attractive candidate for visible-light-driven photocatalysis. To further improve its efficiency, researchers have explored the construction of heterojunction photocatalysts, which promote charge separation and suppress electron–hole recombination. For instance, Pahi et al. demonstrated that combining Cu_2_O with Ag_3_PO_4_ in a p–n heterojunction structure resulted in excellent photocatalytic activity [108]. Expanding on this concept, Doan et al. developed Cu_2_O/Ag_2_MoO_4_ nanoheterojunctions and immobilized them onto cellulose fibers (CFs) sourced from discarded cigarette butts, providing both a sustainable substrate and enhanced photocatalytic performance. The strong solid–solid interface formed between Cu_2_O and Ag_2_MoO_4_ on the CFs facilitated efficient charge transfer, thereby minimizing electron–hole recombination and creating favorable conditions for complex photochemical reactions during the degradation of Reactive Blue 19 dye. This hybrid design not only improved photocatalytic efficiency but also enhanced the durability and reusability of the catalyst, underlining the value of cellulose-based supports in stabilizing heterojunction photocatalysts for wastewater treatment [109].

Using cellulose as a support matrix for photocatalytic processes offers multiple advantages that make it highly attractive for sustainable applications. As a natural, renewable, and biodegradable material, cellulose is not only eco-friendly but also widely available and low in cost, making it a practical choice compared to synthetic supports. Structurally, it provides a large surface area and a highly interconnected network, particularly in forms such as BC, nanocellulose, and MFC, which create ideal platforms for anchoring photocatalytic nanoparticles like ZnO, TiO_2_, Cu_2_O, or hybrid nanocomposites. The abundance of functional groups such as hydroxy, carboxyl, and amidoxime enables strong interactions with metal ions, preventing nanoparticle aggregation and ensuring their uniform dispersion across the cellulose matrix. This stable distribution increases the number of accessible active sites, thereby enhancing photocatalytic efficiency. Beyond serving as support, cellulose itself contributes to pollutant removal through adsorption, creating a synergistic effect that further boosts degradation performance. Moreover, cellulose matrices provide mechanical stability and facilitate reusability, allowing photocatalysts to maintain consistent activity across multiple treatment cycles. Together, these unique features highlight cellulose as an ideal, multifunctional support for the design of efficient, durable, and eco-friendly photocatalytic systems for environmental remediation.

## Conclusion

In conclusion, cellulose stands out as a highly valuable and sustainable material that can be obtained from diverse natural and waste sources, including plants, agricultural residues, and even unconventional waste like discarded cigarette butts. Its abundance and low cost make it an attractive option for large-scale use. Through various chemical processes, such as acid hydrolysis, alkaline treatment, and other advanced techniques, cellulose can be extracted and refined into different forms, ranging from purified cellulose to nanocellulose and MFC. These advanced forms provide high surface area and abundant functional groups, enabling broad applicability across fields such as biomedicine, water treatment, packaging, and energy storage. Altogether, cellulose and its derivatives represent an important foundation for developing next-generation sustainable materials and technologies.

One of the most promising applications of cellulose lies in its use as a support matrix for photocatalytic systems. Its unique structure provides an ideal platform for stabilizing photocatalytic nanoparticles, preventing their aggregation, and ensuring better dispersion throughout the matrix. At the same time, cellulose can adsorb pollutants directly from water, creating a synergistic effect that enhances the overall degradation process. These combined features significantly improve photocatalytic efficiency and reliability. Numerous studies have demonstrated that cellulose-based composites, particularly when integrated with photocatalysts such as ZnO, TiO_2_, or Cu_2_O, which can effectively degrade harmful dyes and other organic pollutants under various light conditions. Beyond their high efficiency, these composites also exhibit strong mechanical stability and excellent reusability, making them practical and sustainable materials for repeated water treatment applications.

Overall, cellulose contributes not only to green and sustainable design but also to real functional improvements in photocatalytic processes. By serving as both a structural support and an active participant in pollutant removal, it enhances the stability, efficiency, and reusability of photocatalysts. These unique qualities make cellulose an excellent and versatile material for advancing environmental cleanup technologies and supporting the global shift toward more sustainable solutions.

## Data Availability

Data sharing is not applicable as no new data was generated or analyzed in this study.
